# Emerging Risks of Amanita Muscaria: Case Reports on Increasing Consumption and Health Risks

**DOI:** 10.15388/Amed.2025.32.1.23

**Published:** 2025-02-18

**Authors:** Emilija Savickaitė, Gabija Laubner-Sakalauskienė

**Affiliations:** 1 Vilnius University, Faculty of Medicine, Vilnius, Lithuania; 2 Centre of Toxicology, Republican Vilnius University Hospital; Faculty of Medicine, Vilnius University, Clinic of Anaesthesiology and Intensive Care, Vilnius, Lithuania

**Keywords:** Amanita muscaria, muscimol, ibotenic acid, microdosing, psychoactive mushrooms, *Amanita muscaria*, muscimolis, iboteno rūgštis, mikrodozavimas, psichoaktyvūs grybai

## Abstract

**Introduction:**

The increasing popularity of *Amanita muscaria*, driven by its hallucinogenic properties, has raised significant public health concerns, particularly as it remains largely unregulated across most European Union countries. The mushroom contains muscimol, a compound that induces euphoria, altered perception, and hallucinations, and its precursor, ibotenic acid, converts to muscimol when dried or heated, reducing toxicity while preserving psychoactive effects. The growing trend in intentional consumption of *A. muscaria* reflects evolving patterns of intoxication despite its known toxicity risks. The *European Food Safety Authority* has flagged *A. muscaria* as an emerging risk, highlighting concerns over its increasing availability and potential for misuse.

**Materials and Methods:**

Four cases of *Amanita muscaria* consumption and subsequent intoxication have been documented in Lithuania in 2023. To further investigate this topic, a systematic search was conducted using the *PubMed* database with the following keyword combinations: ‘*Amanita muscaria’*, ‘*Amanita muscaria* toxicity’, ‘muscimol’, ‘ibotenic acid’, ‘psilocybin’, and ‘hallucinogenic fungi’. After screening for relevance and eligibility, a total of 27 publications met the inclusion criteria and were incorporated into the final analysis.

**Case Reports:**

In 2023, four cases of intentional *A. muscaria* poisoning were reported in Lithuania, linked to recreational consumption. Symptoms included tremors, respiratory failure, dizziness, and paranoia. All patients were male and required hospitalization, but all were discharged in stable condition.

**Conclusion:**

The unregulated status and increasing accessibility of *A. muscaria* pose significant public health concerns. While *A. muscaria* remains largely unstudied in medical contexts, its toxicity risks are well-documented. Misleading online information contributes to uninformed consumption, especially among younger individuals. Further research is needed to elucidate its chemical composition, therapeutic potential, and health effects to inform regulatory policies.

## Abstract (it)

**Įvadas:**

*Amanita muscaria* (paprastoji musmirė) – musmirių (Amanita) genties nuodingas grybas – populiarumas dėl haliucinogeninių savybių kelia didelį susirūpinimą dėl pavojaus visuomenės sveikatai, ypač dėl to, kad daugelyje Europos Sąjungos šalių jo vartojimas iš esmės nereglamentuojamas. Grybo sudėtyje yra muscimolio, junginio, kuris sukelia euforiją, pakitusį suvokimą ir haliucinacijas, o jo pirmtakas – iboteno rūgštis – džiovinant ar kaitinant virsta muscimoliu, todėl sumažėja toksiškumas, tačiau išlieka psichoaktyvus poveikis. Didėjanti *A. muscaria* tyčinio vartojimo tendencija atspindi besikeičiančius apsinuodijimo modelius, nepaisant žinomo toksiškumo pavojaus. Europos maisto saugos tarnyba pažymėjo, kad *A. muscaria* kelia naują pavojų, ir išreiškė susirūpinimą dėl didėjančio jo prieinamumo ir piktnaudžiavimo juo galimybių.

**Medžiagos ir metodai:**

2023 m. Lietuvoje užfiksuoti keturi *Amanita muscaria* vartojimo ir vėlesnio apsinuodijimo atvejai. Siekiant išsamiau ištirti šią temą, buvo atlikta sisteminė paieška naudojant *PubMed* duomenų bazę su šiais raktažodžių deriniais: „Amanita muscaria“, „Amanita muscaria toxicity“, „muscimol“, „iboteno rūgštis“, „psilocibinas“ ir „haliucinogeniniai grybai“. Atlikus aktualumo ir tinkamumo atranką, įtraukimo kriterijus atitiko 27 publikacijos, jos ir buvo įtrauktos į galutinę analizę.

**Atvejai:**

2023 m. Lietuvoje užregistruoti keturi tyčinio apsinuodijimo *A. muscaria* atvejai, susiję su pramoginiu vartojimu. Simptomai buvo drebulys, kvėpavimo nepakankamumas, galvos svaigimas ir paranoja. Visi pacientai buvo vyrai, juos reikėjo hospitalizuoti, tačiau visi buvo išrašyti stabilios būklės.

**Išvada:**

*A. muscaria* nereguliuojamas ir vis labiau prieinamas vartojimas kelia didelį susirūpinimą visuomenės sveikatai. Nors *A. muscaria* iš esmės nėra ištirta medicininiu požiūriu, jos toksiškumo pavojus yra gerai dokumentuotas. Klaidinanti informacija internete prisideda prie neinformuoto vartojimo, ypač tarp jaunesnių žmonių. Siekiant išsiaiškinti jos cheminę sudėtį, gydomąjį potencialą ir poveikį sveikatai, reikia tolesnių tyrimų, kad būtų galima pagrįsti reguliavimo politiką.

## Introduction

The increasing demand for *Amanita muscaria*, commonly known as fly agaric, due to its hallucinogenic properties has raised concerns, particularly as it remains largely unregulated in most European Union countries, in contrast to psychoactive psilocybin-containing mushrooms. The *Amanita* genus, one of the most extensively studied fungal families, comprises approximately 600 described species within the order *Agaricales* and includes several highly toxic species responsible for the majority of fatal mushroom poisonings [1,2]. Its psychoactive effects are primarily attributed to muscimol, a compound known to induce euphoria, altered sensory perception, dizziness, and hallucinations. Ibotenic acid, the metabolic precursor to muscimol, undergoes decarboxylation through drying or heating, thereby reducing its toxicity while preserving its psychoactive properties. The growing use of *Amanita muscaria* for its psychoactive effects reflects an evolving trend in substance use, raising concerns regarding potential health risks despite its perceived therapeutic applications.

Although *Amanita muscaria* exists within a regulatory ‘gray area’ among psychoactive substances, its legal ambiguity situates it within the category of *New Psychoactive Substances* (NPS) in certain contexts. While NPS are predominantly synthetic, the classification also encompasses naturally occurring compounds that circumvent international drug control conventions. According to the European Drug Report 2024, approximately 50 new psychoactive substances entered the market annually between 2016 and 2022, with 26 new substances identified in 2023 alone [3]. Furthermore, the 2023 European Food Safety Authority report raised concerns regarding the increasing prevalence of alcohol replacement products derived from food-grade herbs that interact with the *Gamma-AminoButyric Acid* (GABA) system. This report also highlighted the rising consumption of *Amanita muscaria* as an emerging public health risk [4]. Given the growing accessibility of such substances through online markets, the potential health risks associated with *Amanita muscaria* necessitate further scientific investigation.

## Methods

Four cases of *Amanita muscaria* consumption and subsequent intoxication have been documented in Lithuania in 2023. To further investigate this topic, a systematic search was conducted using the *PubMed* database with the following keyword combinations: ‘Amanita muscaria’, ‘Amanita muscaria toxicity’, ‘muscimol’, ‘ibotenic acid’, ‘psilocybin’, and ‘hallucinogenic fungi’. The search was limited to peer-reviewed, full-text articles published in English, while non-full-text and non-English publications were excluded. After screening for relevance and eligibility, a total of 27 publications met the inclusion criteria and were incorporated into the final analysis.

## Case reports

Historically, the majority of *Amanita muscaria* intoxication cases were attributed to misidentification or confusion with other mushroom species. However, in 2023, all four reported poisoning incidents in Lithuania resulted from intentional consumption of *Amanita muscaria* for recreational purposes. This shift in the etiology of poisoning underscores a growing trend in the deliberate use of *Amanita muscaria* for its psychoactive effects, reflecting a significant change in intoxication patterns.

Among the reported cases, one patient developed tremors, hyperhidrosis, and respiratory failure requiring intubation and mechanical ventilation after ingesting three dried mushrooms. Another patient, who consumed two mushrooms as part of a bet, presented with dizziness, generalized weakness, and nausea. A third case involved a patient with a history of substance use who exhibited agitation, paranoia, and disorientation, necessitating sedation with antipsychotics before achieving full recovery. The fourth patient, who ingested a homemade mushroom infusion, displayed euphoria and disorientation. All affected individuals were male, representing a broad age range with no specific age group predominating. Each patient required hospitalization and, following appropriate medical intervention, was discharged in stable condition.

## Discussion

Recently, an increasing number of European countries have begun to impose restrictions on *Amanita muscaria*. In Lithuania, the mushroom has been classified as a controlled substance under the List of Narcotic and Psychotropic Substances, with the ban taking effect at the beginning of 2025 [5]. Similarly, the Estonian government has been drafting legislation aimed at curbing the promotion of hazardous pseudo-medicinal products, including the *Miracle Mineral Supplement* (MMS) and toxic *Amanita* mushrooms. This proposed law, designed to grant the Estonian Health Board (Terviseamet) the authority to monitor and penalize individuals and entities promoting these substances, was scheduled to take effect on January 1, 2025. However, no further information regarding its implementation has been made publicly available [6]. Within the European Union, *Amanita muscaria* has already been banned in Romania and the Netherlands, while its commercial sale is prohibited in Poland [7–9]. Poland is among the few countries where policymakers have addressed the rising popularity and risks of *Amanita muscaria*, expressing concern over its accessibility. Despite its recognized toxicity and the absence of approved medicinal products, the mushroom remains readily available for purchase through online marketplaces across the European Union. These developments have sparked debates on its regulation and potential applications [10]. Advocates for its use often cite its historical role in religious rituals, particularly among Indigenous Siberian shamans, to support claims of its potential benefits [11]. While historical and cultural heritage provides important context for discussions on psychoactive substances, some proponents attempt to draw parallels between *Amanita muscaria* and psilocybin-containing mushrooms, suggesting potential medicinal applications [12].

The psychoactive effects of *Amanita muscaria* are primarily attributed to muscimol, a highly selective agonist of ionotropic GABA_A_ receptors [13]. By acting on the cerebral cortex, thalamus, hippocampus, and cerebellum, muscimol mimics the inhibitory effects of GABA_A_, resulting in euphoria, dizziness, heightened sensory perception, coordination impairment, and visual hallucinations [14,15]. Amanita muscaria also contains ibotenic acid, a metabolic precursor to muscimol. While ibotenic acid itself is not classified as a psychoactive compound, both muscimol and ibotenic acid exhibit toxic properties. Proper preparation methods, such as drying or heating, facilitate the decarboxylation of ibotenic acid into muscimol, thereby reducing toxicity while preserving psychoactive effects [14]. Notably, other substances with a higher potential for addiction, such as benzodiazepines, barbiturates, and alcohol, exert similar modulatory effects on GABA_A_ receptors [16]. Several bioactive compounds have been identified in *Amanita muscaria*, including muscarone, choline, acetylcholine, betaine, muscarine, hyoscyamine, atropine, scopolamine, and bufotenine [17]. However, their combined effects on the central nervous system remain insufficiently understood. Further research is necessary to elucidate the interactions among these compounds, particularly as processed *Amanita muscaria* products – such as tinctures, powders, and gummies – are increasingly available through online markets. A study found that trehalose in commercial *Amanita muscaria* extracts significantly increased IL-8 levels and pro-inflammatory cytokine production in brain cells [18]. While the mechanism is still unclear, this suggests that trehalose may modulate microglial activity and neuroinflammation. It is also linked to enhanced autophagy and reduced neurotoxicity, highlighting its therapeutic potential [19,20]. Given the growing interest and availability of *Amanita muscaria* preparations, unbiased research is needed to assess compound variations and the potential toxicity in both synthesized and natural formulations. Future studies should prioritize independent, unfunded research to avoid conflicts of interest.

The widespread dissemination of information regarding the potential medicinal properties of *Amanita muscaria* has led to frequent comparisons with psilocybin-containing mushrooms. While psilocybin mushrooms were once explored for psychiatric treatment, their use was largely abandoned following the adoption of the 1971 United Nations Convention on Psychotropic Substances, which imposed strict regulatory bans in European countries [21]. However, over the past decade, research interest in the therapeutic potential of psilocybin has grown substantially, often referred to as a “renaissance in psychedelic research” [22]. A bibliometric analysis of human studies on psychedelics revealed that 54% of the top 100 most-cited articles were published between 2010 and 2020, reflecting a significant resurgence of scientific inquiry into this area [23]. The renewed interest in psilocybin-containing mushrooms is largely attributed to their mechanism of action, primarily through the activation of 5-HT_2A_ receptors, which may be associated with antidepressant, anxiolytic, and neuroplastic effects [24]. Recent studies show that psilocybin modulates brain activity by increasing and decreasing blood flow in different regions, with heightened activity in the frontal cortex and reduced perfusion elsewhere [25,26]. These effects are central to its potential in psychedelic-assisted therapy, where controlled use may offer therapeutic benefits [27]. Psilocybin also influences brain networks, particularly the *Default Mode Network* (DMN), involved in self-referential thought, enhancing connectivity and disrupting rigid cognitive patterns. This contributes to its efficacy in treating conditions such as depression, *Obsessive-Compulsive Disorder* (OCD), and *Post-Traumatic Stress Disorder* (PTSD). However, outside controlled settings, psilocybin can trigger adverse effects, particularly in individuals with psychiatric disorders like schizophrenia, where it may induce psychosis [28].

Despite frequent associations with psilocybin-containing mushrooms, *Amanita muscaria* and its primary active compound, muscimol, do not interact with serotonin 5-HT_2A_ receptors. The psychoactive effects of *Amanita muscaria* are often misinterpreted as therapeutic or beneficial, although they are primarily attributed to muscimol’s modulation of the GABA_A_ receptor system [11,12,29]. The tendency among users to perceive *Amanita muscaria* as a therapeutic substance reflects a widespread misconception that its effects may parallel those of psilocybin mushrooms. A recent study analyzed survey responses from individuals active in Facebook groups dedicated to *Amanita muscaria* consumption, where users frequently share information and personal experiences related to its use [12]. The findings indicated that motivations for consumption were associated with both physical and mental well-being, though notable gender differences were observed. Among female respondents, 85.7% reported using *Amanita muscaria* for skin-related issues, while 58.7% consumed it for pain relief. In contrast, 77.8% of male respondents cited stress relief as their primary reason for use, followed by 74.5% for alleviating depressive symptoms and 64.3% for managing insomnia. Another large-scale study investigated the medicinal use of fungi across eight former Soviet Union republics by comparing historical records with contemporary ethnographic interviews [29]. The study found that 67% of the respondents held a positive perception of the medicinal applications of fungi within these regions. Despite the toxic nature of *Amanita muscaria*, it played a significant role in traditional practices, with users frequently highlighting its purported benefits [11,29]. Many interviewees expressed a strong belief in its historical use as a fly repellent, a function that is reflected in the mushroom’s name across multiple languages within the studied regions. While acknowledging its toxic properties, respondents also emphasized its perceived efficacy in traditional medicine, underscoring a longstanding cultural belief in its medicinal applications [29].

While psychedelic microdosing – typically involving sub-threshold doses of serotonergic psychedelics to enhance cognitive and emotional well-being – has gained significant scientific and public attention, the practice of microdosing *Amanita muscaria* remains largely unexplored [30]. Ethnomycology, the study of fungi’s historical uses and sociocultural impact, provides valuable insights into traditional consumption practices, including microdosing. The influence of ethnomycological knowledge is evident in both physical and online literature, where custodians of folk traditions document the historical applications of *Amanita muscaria*. Research suggests that ethnomycological knowledge passed down through familial traditions remains a reliable source of information for many users [29]. Despite growing interest, no scientifically rigorous studies on *Amanita muscaria* microdosing have been published in peer-reviewed journals, rendering all claims regarding its potential benefits in popular literature still unverified. However, several preclinical studies have investigated the effects of *Amanita muscaria*’s primary psychoactive compounds, namely, muscimol and ibotenic acid [31–36]. These studies primarily examine their interactions with GABA receptors in the central nervous system, suggesting potential sedative properties while also indicating possible roles in neuroprotection, cognitive enhancement, and endocrine modulation [31–36]. Although preclinical findings provide initial insights into the pharmacological effects of muscimol and ibotenic acid, clinical trials are necessary to confirm or disprove their therapeutic potential and establish evidence-based applications.

## Conclusion

The increasing accessibility and largely unregulated status of *Amanita muscaria* raise significant public health concerns, particularly regarding the spread of misinformation. Unlike psilocybin, which is currently undergoing clinical investigation for its potential therapeutic applications, *Amanita muscaria* remains largely unstudied in a medical context and presents notable toxicity risks. The widespread dissemination of misleading information, particularly through online marketplaces, contributes to uninformed consumption, posing potential health risks, especially among younger demographics. Strengthening regulatory oversight and public education initiatives is essential to mitigate these risks.

Recent poisoning cases involving *Amanita muscaria* are increasingly attributed to intentional use rather than accidental ingestion, thus reflecting a shift in consumption patterns. This trend suggests a growing interest in the mushroom’s psychoactive effects, raising concerns regarding misuse, toxicity, and the persistence of misconceptions about its safety. While preclinical studies indicate that muscimol and ibotenic acid may exert neuroactive effects, their therapeutic potential remains unverified due to the absence of clinical trials. Further research is necessary to elucidate the chemical composition, dosing parameters, and health implications of *Amanita muscaria*. Comprehensive scientific understanding is essential for informing future policy decisions and clinical evaluations, while ensuring that any potential applications are based on robust empirical evidence.
